# Moderate Salinity Stress Affects Rice Quality by Influencing Expression of Amylose- and Protein-Content-Associated Genes

**DOI:** 10.3390/ijms25074042

**Published:** 2024-04-05

**Authors:** Chongke Zheng, Shulin Niu, Ying Yan, Guanhua Zhou, Yongbin Peng, Yanan He, Jinjun Zhou, Yaping Li, Xianzhi Xie

**Affiliations:** 1 Institute of Wetland Agriculture and Ecology, Shandong Rice Engineering Technology Research Center, Shandong Academy of Agricultural Sciences, Jinan 250100, China; zhengck1983@163.com (C.Z.); nsl199811@163.com (S.N.); xichenzgh@163.com (G.Z.); daluo1210@outlook.com (Y.P.); hyn2013nice@163.com (Y.H.); lyp8908@126.com (Y.L.); 2 Crop Breeding and Cultivation Research Institute, Shanghai Academy of Agricultural Sciences, Shanghai 201403, China; yanying04@163.com; 3 Institute of Crop Germplasm Resources, Shandong Academy of Agricultural Sciences, Jinan 250100, China; zhousdrice@163.com

**Keywords:** rice, salinity, amylose content, grain protein content, quality-associated gene

## Abstract

Salinity is an environmental stress that severely impacts rice grain yield and quality. However, limited information is available on the molecular mechanism by which salinity reduces grain quality. In this study, we investigated the milling, appearance, eating and cooking, and nutritional quality among three japonica rice cultivars grown either under moderate salinity with an electrical conductivity of 4 dS/m or under non-saline conditions in a paddy field in Dongying, Shandong, China. Moderate salinity affected rice appearance quality predominantly by increasing chalkiness rate and chalkiness degree and affected rice eating and cooking and nutritional quality predominantly by decreasing amylose content and increasing protein content. We compared the expression levels of genes determining grain chalkiness, amylose content, and protein content in developing seeds (0, 5, 10, 15, and 20 days after flowering) of plants grown under saline or non-saline conditions. The chalkiness-related gene *Chalk5* was up-regulated and *WHITE-CORE RATE 1* was repressed. The genes *Nuclear factor Y* and *Wx,* which determine amylose content, were downregulated, while protein-content-associated genes *OsAAP6* and *OsGluA2* were upregulated by salinity in the developing seeds. These findings suggest some target genes that may be utilized to improve the grain quality under salinity stress conditions via gene-pyramiding breeding approaches.

## 1. Introduction

As one of the most important staple crops, rice (*Oryza sativa* L.), provides food for more than half of the world’s population. However, rice is a salt-sensitive crop and salt stress seriously affects the yield and quality of rice [1,2]. Rice quality includes many aspects, such as milling, appearance, eating and cooking, and nutritional quality. The milling quality includes the brown rice rate (BRR, %), milled rice rate (MRR, %), and head milled rice rate (HMRR, %). The appearance quality includes the grain length, grain width, and chalkiness. The eating and cooking quality (ECQ) mainly includes the fragrance, hardness, viscosity, and degree of balance. The nutritive quality includes the amylose content, protein content, fat content, and gel consistency of rice grain [3]. These characteristics are the main factors determining the taste quality, the market price, consumers’ choices, and commercial prospects [4].

The molecular basis underlying rice grain appearance is well known. Grain shape, including grain length, grain width, and aspect ratio, is controlled by multiple genes, e.g., *GW2* [5], *GW6a* [6], *GS3* [7], *DEP1* [8], *GW5* [9], and *GL7* [10]. Chalkiness is one of the important indicators of rice appearance quality, and the level of chalkiness determines the commerciality of rice. Up to now, only two QTLs related to chalkiness, a vacuolar H^+^ translocating pyrophosphatase-encoding locus *Chalk5* and a F-box protein-encoding locus *WHITE-CORE RATE 1 (WCR1)*, have been cloned [11,12]. Elevated expression of *Chalk5* causes chalky grains [11], whereas *WCR1* functions to reduce chalkiness [12].

Grain amylose content (AC) is a key factor determining the ECQ and nutritional quality of rice grains [13]. Amylose content is an important quality of rice. If the amylose content is too high (>25%), the cooked rice tastes hard, loose, and poor. When the amylose content is too low (<2%), the cooked rice tastes too soft, sticky, and poor [13]. The *Waxy* (*Wx*) gene, encoding the enzyme Granule Bound Starch Synthase I (GBSSI), functions to regulate amylose content in cereal endosperm [14].

Protein is the most important storage substance in rice seed except starch, and its content and composition are crucial for the ECQ and nutritional value of rice [15]. At present, only two QTLs related to stable grain protein content (GPC) have been successfully identified and cloned. Peng et al. cloned a QTL (*qGC1*) affecting protein content for the first time, which encodes an amino acid transporter amino acid permease 6 (OsAAP6) [16]. *OsGluA2*, encoding a glutelin type-A2 precursor, functions as a positive regulator of GPC [17].

Although it has been reported that salinity reduced rice yield, limited information about the effect of salinity on rice quality, as well as the underlying molecular bases, has been reported. In this study, the quality traits of three japonica rice cultivars grown either under moderate salinity (with an EC of 4 dS/m) or under non-saline conditions in a paddy field were investigated and analyzed. The results showed that salinity causes a reduction in grain AC and an increase in GPC and chalkiness. We analyzed the expression of genes related to AC, GPC, and chalkiness. We report for the first time about the effects of salinity on the expression levels of genes that determine japonica rice grain quality. These findings provide useful information to help breeders develop high-quality salt-tolerant japonica rice cultivars.

## 2. Results

### 2.1. Moderate Salinity Has No Effect on Rice Milling Quality but Reduces Appearance Quality

**Figure 1:**
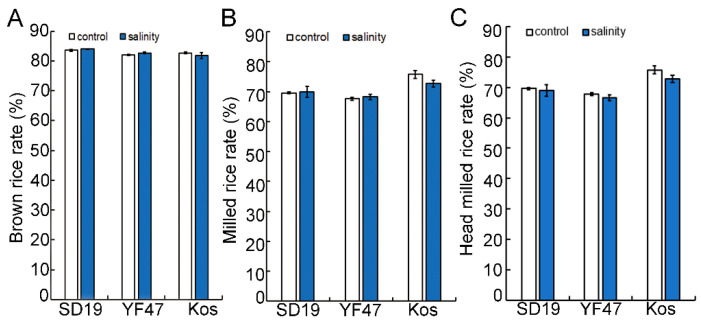
Effects of moderate salinity on rice milling quality in three rice cultivars. (**A**–**C**) Brown rice rate (BRR, %), milled rice rate (MRR, %) and head milled rice rate (HMRR, %) of ‘Shengdao 19′ (SD19), ‘Yanfeng 47’ (YF47) and ‘Koshihikari’ (Kos) under the non-saline condition (control) or moderate salinity with electrical conductivity of 4 dS/m.

To investigate the effect of salinity on the grain milling quality, the BRR, MRR, and HMRR were measured in rice grains from three cultivars grown under either moderate salinity (with an EC of 4 dS/m) or non-saline conditions (control). The values of BRR and MRR had no significant difference between grains from non-saline conditions and from saline conditions in these three japonica cultivars (Figure 1). The HMRR in all three materials decreased under salt stress, but the results are not significant compared to the non-saline condition. These results indicate that salinity stress had no significant effect on the rice milling quality of the three rice cultivars.

**Figure 2:**
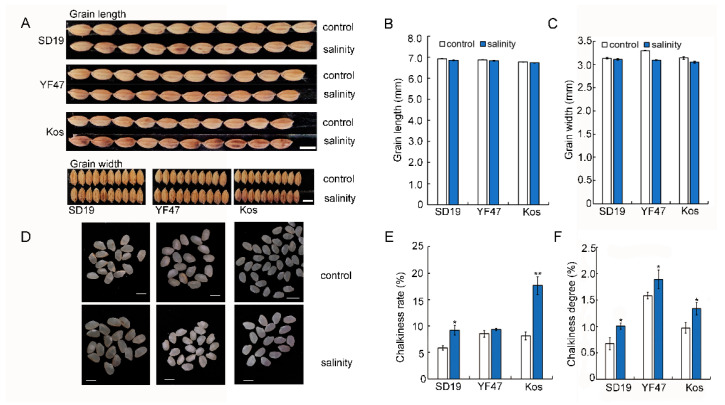
Effects of moderate salinity on appearance quality in three rice cultivars. (**A**,**D**) Grain and polished rice shape of SD19, YF47, and Kos plants treated with moderate salinity and the control. Scale bar = 5 mm. (**B**,**C**) Grain length and width of plants treated with moderate salinity and the control. (**E**,**F**) Chalkiness rate and chalkiness degree of plants treated with moderate salinity and the control. Significant differences were determined with Student’s *t* test or the Mann–Whitney–Wilcoxon test according to the normal distribution of the data (** *p* < 0.01, * *p* < 0.05). Values are the mean ± standard error from 20 individual plants.

In the previous study, we found that moderate salinity stress did not affect grain length and width [18]. In this study, we investigated the grain appearance, including the chalkiness rate and chalkiness degree, of SD19, YF47, and Koshihikari grown under either the control or salinity stress. Compared with those from the control plants, grains from plants grown under salinity stress showed a significantly increased chalkiness rate by 57.14%, 9.80%, and 116.32%, respectively (Figure 2E). Similarly, the chalkiness degree of grains from SD19, YF47, and Koshihikari plants grown under moderate salinity were increased by 49.93%, 19.32%, and 37.80%, respectively (Figure 2F). These results suggest that moderate salinity increased grain chalkiness rate and chalkiness degree.

### 2.2. Moderate Salinity Reduced Amylose Content but Increased Protein Content

**Figure 3:**
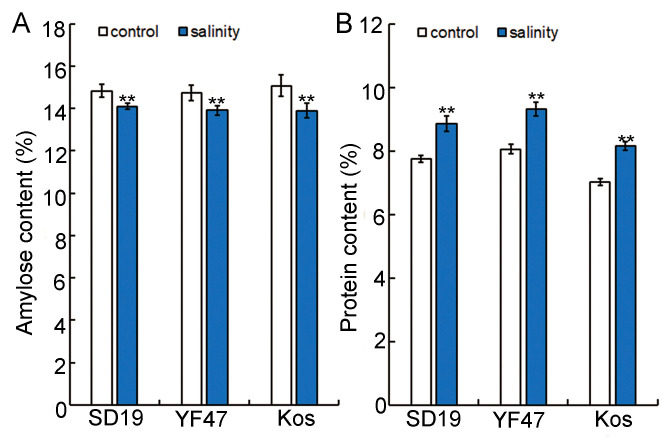
Effects of moderate salinity on amylose and protein content in three rice cultivars. (**A**) Amylose content of SD19, YF47, and Kos plants treated with moderate salinity and the control. (**B**) Protein content of SD19, YF47, and Kos plants treated with moderate salinity and the control. Significant differences were determined with Student’s *t* test or the Mann–Whitney–Wilcoxon test according to the normal distribution of the data (** *p* < 0.01). Values are the mean ± standard error from three measurements.

Amylose content (AC) is an important index to determine the taste value of rice [4], so we conducted amylose content determination on the SD19, YF47, and Koshihikari varieties. The results showed that the amylose content of rice under salt stress was significantly decreased, and that of SD19, YF47, and Koshihikari was decreased by 4.94%, 5.59%, and 7.83%, respectively, compared with the control (Figure 3A).

Grain protein content (GPC) is considered to be an important nutritional factor of rice, and the level of protein content also has a certain impact on food quality [15]. Therefore, the protein contents of SD19, YF47, and Koshihikari were determined under control and moderate salt stress. The results showed that the protein content of rice grain under salt stress increased significantly by 12.41%, 13.57%, and 13.88% in SD19, YF47, and Koshihikari, respectively, compared with the control (Figure 3B). These results indicate that moderate salt stress may influence the taste value of rice by decreasing the amylose content and increasing the protein content.

### 2.3. Moderate Salinity Affected Expression Patterns of Genes That Determine Amylose and Protein Content

**Figure 4:**
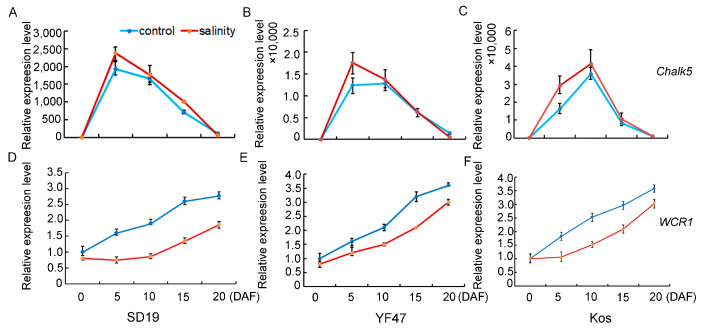
Relative expression levels of genes that determine chalkiness under moderate salinity in SD19 (**A**,**D**), YF47 (**B**,**E**), and Kos (**C**,**F**). Expression levels were determined in seeds at 0, 5, 10, 15, and 20 days after flowering (DAF) under moderate salinity with an EC of 4 dS/m and the non-saline (control) condition.

In order to study the molecular basis of how moderate salt stress affects rice quality, the expression levels of genes that determine chalkiness rate, AC, and GPC were detected and compared in the developing seeds at 0, 5, 10, 15, and 20 days after flowering (DAF) in SD19, YF47, and Koshihikari grown either under moderate salt 4 dS/m stress or under the non-saline condition. *Chalk5*, the major QTL for controlling chalkiness, was significantly upregulated in the seeds at the 5th and 10th day after flowering under salt stress conditions (Figure 4), which was consistent with the increased chalkiness rate observed under salinity stress. *WCR1*, which negatively regulates grain chalkiness and improves grain quality in rice, was significantly repressed by salinity. These results suggest that salt stress regulates chalkiness by upregulating *Chalk5* expression and downregulating *WCR1* expression.

**Figure 5:**
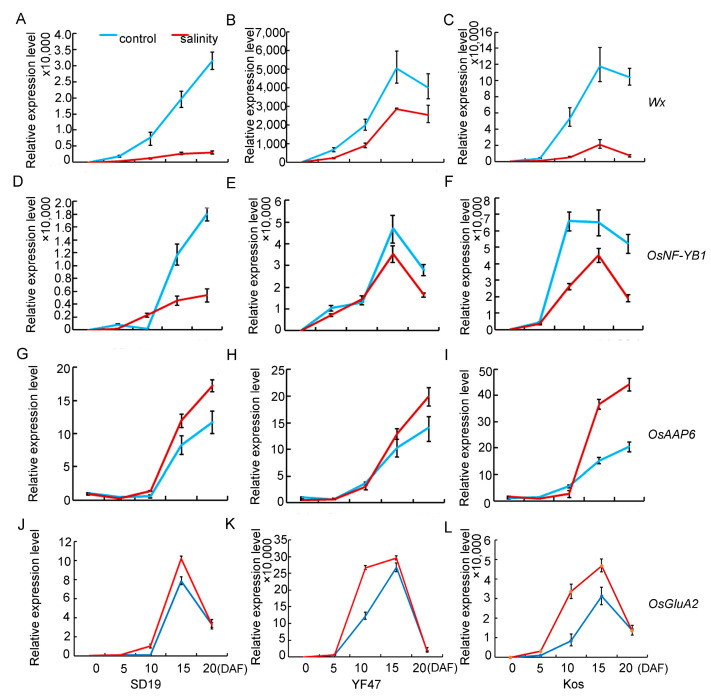
Relative expression levels of genes that determine AC (*Wx* and *OsNF*-*YB1*) and GPC (*OsAAP6* and *OsGluA2*) under moderate salinity in SD19 (**A**,**D**,**G**,**J**), YF47 (**B**,**E**,**H**,**K**), and Kos (**C**,**F**,**I**,**L**). Expression levels were determined in seeds at 0, 5, 10, 15, and 20 days after flowering (DAF) under moderate salinity with an EC of 4 dS/m and the non-saline (control) condition.

Given that salinity stress affects AC and GPC, the expression levels of *Wx*, *OsNF-YB1*, and *OsAAP6* genes, which are associated with AC and GPC, were compared in seeds at 0, 5, 10, 15, and 20 DAF under moderate salt (4 dS/m) stress or under the control. The results showed that transcript levels of *Wx* were significantly decreased from 5 DAF under salinity stress (Figure 5A–C) compared with those of the non-saline plants. The expression level of *OsNF-YB1* was severely decreased under salinity stress from 10 DAF (Figure 5D–F). Contrary to the expression pattern of *Wx* and *OsNF-YB1*, the expression of the gene *OsAAP6* for regulating GPC was increased from 10 DAF. These results suggested that salinity stress decreased AC by repressing the expression of the *Wx* and *OsNF-YB1* genes associated with starch synthesis, but positively regulated GPC by improving the expression of the *OsAAP6* and *OsGluA2* genes associated with protein synthesis and accumulation.

### 2.4. Salinity Stress Probably Regulates Quality-Associated Genes’ Expression through Stress-Related Cis-Elements

**Figure 6:**
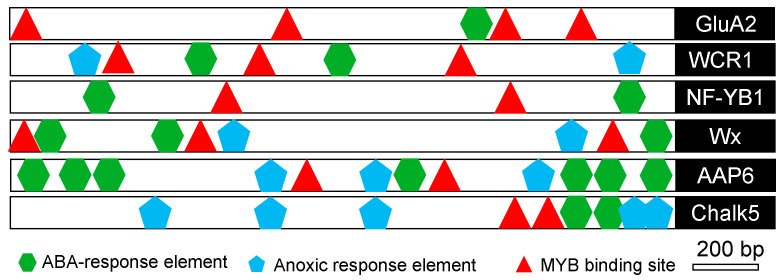
In silico analysis of the putative promoter region of stress-regulated quality-associated genes in rice. The diagram shows the approximate positions of putative stress-related *cis*-regulatory elements present in the ~2 kb upstream region of salinity-regulated quality-associated genes in rice as predicted by the PlantCARE database. MYB-binding sites (MBS), anoxia-responsive elements (ARE), and abscisic acid-responsive elements (ABRE) are represented by different symbols.

In order to analyze the molecular basis of the aforementioned quality genes in response to salt stress, *cis*-elements were analyzed using the PlantCARE database in the 2 kb sequence upstream of each gene [19]. The results showed that several stress-related *cis*-elements, such as abscisic acid responsive elements (ABRE), anoxic-responsive elements (ARE), and MYB-binding sites (MBS), were predicted to exist in all the aforementioned genes (Figure 6). These results implied that salt stress regulated the expression of the genes associated with quality.

## 3. Discussion

Environmental stresses not only affect milling yield, but also significantly affect the cooking quality. However, the molecular mechanism of salt stress affecting rice quality is still unclear. In this study, we validated that japonica rice quality was affected under moderate salinity (with an EC of 4 dS/m) by increasing chalkiness and the grain protein content and reducing the grain amylose content. Similar observations have been reported [20,21,22,23,24]. However, different results were reported by Thitisaksakul et al. [25] and Sangwongchai et al. [2], in which the amylose content and protein content had no significant changes under salinity stress. The difference probably results from the different salt tolerances of the rice cultivars used in individual studies. In addition, the period of salt treatment in our study is from the seedling stage until harvest, while in Sangwongchai’s report, it was from the anthesis stage until harvest [2]. In addition, the saline water used in our study was diluted underground water with pH 8.0, while in Thitisaksakul’s report, 20 mM or 40 mM NaCl was added to water to achieve EC values of 2 and 4 dS/m [25].

It has been reported that salinity significantly increased the BRR and MRR [4]. However, in our study, we observed that the BRR and MRR have no significant changes under moderate salinity (Figure 2A,B). The difference probably results from the salt concentration [4]. In addition, it has been reported that high temperature and drought stress during the grain filling stage led to the decline of rice appearance quality, such as decreased grain length and width and increased chalkiness rate [26,27]. In this study, salt stress did not significantly change the grain shape (Figure 2A) but increased the chalkiness rate (Figure 2E). The expression of the major chalkiness rate gene *Chalk5* was significantly upregulated, whereas *WCR1* was repressed under salt stress (Figure 4). The present results suggest that the upregulated expression of *Chalk5* and repressed expression of *WCR1* in the panicle coordinately increase the rice chalkiness rate under moderate salinity stress.

Starch and protein in the endosperm are the major components of rice grain. Starch is stored in the endosperm in two forms, namely amylose and amylopectin. Amylose and protein content are important factors affecting the ECQ and nutrition of rice [13,15]. In the present study, we observed that the amylose content was significantly decreased and the protein content was significantly increased by salinity (Figure 3), which is consistent with previous reports [20,21,22,23,24]. Consistent with the decreased amylose content and increased protein content, the expression level of the starch synthesis gene *Wx* was repressed, whereas those of the *OsAAP6* and *GluA2* genes were induced by moderate salinity stress in this study (Figure 5). Similar to the results from our study, it had been reported that drought stress and high temperature and low temperature stress inhibited the expression of *Wx* and accelerated the expression of the *OsAAP6* and *GluA2* genes in the early and middle stages of rice grain filling [28,29,30,31,32].

However, the molecular mechanism by which salinity regulates the expression of the *Chalk5*, *Wx*, *OsNF*-*YB1*, *OsAAP6*, and *OsGluA2* genes has not been elucidated. It has been reported that MBS, ARE, and ABRE are stress-related *cis*-elements [33,34,35]. In silico analyses revealed that such stress-related *cis*-elements exist in the promoters of *Chalk5*, *Wx*, *OsNF*-*YB1*, *OsAAP6*, and *OsGluA2* (Figure 6), indicating that these genes are probably regulated by salt stress. The MYB recognition site in the *RD22* promoter region functions as a *cis*-acting element in the drought- and ABA-induced gene expression of *RD22* [36]. Alcohol dehydrogenase 1 (*ADH1*), including the ARE element in its promoter, confers both abiotic and biotic stress resistance in *Arabidopsis* [37,38]. ABRE was found in most stress-induced genes [39]. Although there were multiple stress-related *cis*-elements in the promoters of *Wx* and *OsNF*-*YB1*, their expression was still inhibited. It is speculated that there are other regulatory factors affecting its expression.

## 4. Materials and Methods

### 4.1. Plant Materials and Growth Conditions

Three japonica rice cultivars, ‘Shengdao 19′ (SD19), ‘Yanfeng 47′ (YF 47), and ‘Koshihikari’ (Kos), were used in this study. The growth condition and processing method were based on our previous research [18]. Briefly, seeds were sowed in early May. In early June, the seedlings were transplanted to an irrigated field in Dongying, Shandong Province, China (latitude 38°15′ N; longitude 118°50′ E). Each cultivar was planted in a plot of about 2.5 squares with eight individuals planted in each row at a spacing of 25 × 14 cm with three replications. Saline water with an EC of 4 dS/m was prepared according to Zheng et al. (2021) [8]. About 7 days after transplantation, the salt stress group was irrigated with saline water from the seedling stage to the mature stage. The salinity of the water was monitored every day and adjusted whenever necessary. For the non-saline control, fresh water was used for irrigation. Other management followed the local farmers’ standard.

### 4.2. Determination of Milling Quality

The brown rice rate (BRR, %), milled rice rate (MRR, %) and head milled rice rate (HMRR, %) were measured according to the previous research [4]. The rice seeds were harvested from each treatment and dried naturally till the grain moisture was below 14% [40]. To obtain brown rice, 200 g of a rice sample was passed through a rice huller (JGMJ8098, made by Shanghai Jiading cereals and oils Instrument Co., Ltd., Shanghai, China). To obtain milled rice, the brown rice was passed through a rice polisher twice (CBS2200A satake Japan, Hiroshima, Japan). Head milled rice constituted the grain with a length of 3/4 or more of the whole milled grain separated from 50 g of milled rice, according to the Determination of head rice yield of paddy (GB/T21719-2008) [41]. We followed a previous report to calculate the brown rice rate, milled rice rate, and head milled rice rates as follows [4]:

brown rice rate = brown rice weight/200 × 100%

milled rice rate = milled rice weight/200 × 100%

head milled rice rate = milled rice weight/50 × milled rice rate

The chalkiness rate and chalkiness degree of head milled rice were measured following a previous report [5].

### 4.3. Determination of Amylose and Protein Content

Amylose content was determined by the method described by Kharshiing (2021) [42]. To make standard curves, 100 mg of standard starch (Sigma, Saint Louis, MI, USA) was suspended into a test tube and 1 mL of 95% ethanol and 9 mL of 1M NaOH were added. The suspension was thoroughly mixed and heated in a dry bath at 100 °C for 10 min. A suitable aliquot of the suspension was mixed with 1N acetic acid and I_2_-KI solution and allowed to stand for 20 min at room temperature. The absorbance of the solution at 620 nm was recorded. Three measurements were taken per material to be tested, and the average value was calculated.

Grain protein content was determined by a rice taste analyzer JSWL (Dongfu Jiuheng, Beijing, China).

### 4.4. RNA Isolation and Quantitative Real-Time (qRT)-PCR Analysis

For analysis of the expression level of genes associated with amylose and protein content, young seeds after flowering 0, 5, 10, 15, and 20 days were harvested from SD19, YF47, and Kos plants grown under either salt stress or control conditions and frozen in liquid nitrogen for further RNA isolation.

Total RNA was extracted using RNAiso Plus (TaKaRa, Dalian, China), according to the manufacturer’s protocol. Reverse transcription was performed with the PrimeScript^®^ RT Enzyme Mix I (TaKaRa, Dalian, China) using the extracted RNA. qRT-PCR assays were conducted using SYBR Premix Ex Taq™ (TaKaRa, Dalian, China) according to a previous report [18]. The rice *eEF-1α* gene (AK061464) was used as an internal control to quantify the relative expression level of each target gene [43]. The qRT-PCR primers used in this study are listed in Supplementary Materials Table S1. The results of RT-qPCR were listed in Supplementary Material Table S2.

### 4.5. In Silico Analysis of the Putative Cis-Regulatory Elements of the Stress-Responsive Quality-Associated Genes

For the analysis of the putative *cis*-regulatory elements of the quality-regulated genes, sequences of about ~2 kb of the upstream regions of *OsNF-YB1*, *Chalk5*, *WCR1*, *Wx*, *OsAAP6*, and *OsGluA2* were retrieved using chromosomal coordinates from the Rice Genome Browser (https://rice.plantbiology.msu.edu/cgi-bin/gbrowse/rice/), accessed on 10 August 2022. The putative *cis*-regulatory elements were analyzed by the PlantCARE database (https://bioinformatics.psb.ugent.be/webtools/plantcare/html/), accessed on 10 August 2022 [19].

### 4.6. Data Analysis

All data represent the mean ± SD. The Shapiro–Wilk test was used to assess the normal distribution of all data. The significant difference analyses for normal and non-normal data were carried out by Student’s *t*-test and the Mann–Whitney–Wilcoxon test, respectively. The significance of differences between means was analyzed with Student’s *t*-test (** *p* < 0.01 and * *p* < 0.05).

## 5. Conclusions

In conclusion, the present study is the first to dissect the molecular basis underlying the effect of salinity on grain quality in japonica rice cultivars. Moderate salinity reduces grain quality by regulating associated genes to chalkiness and amylose and protein content, including *Chalk5*, *WCR1*, *OsNB*-*YF1*, *Wx*, *OsAAP6*, and *OsGluA2*. Although how these genes are regulated by salinity requires comprehensive investigation in the future, the present findings suggest some targets that may be utilized to improve grain quality under salinity stress conditions. For example, the *Chalk5* gene can be edited by CRISPR/Cas9 to reduce chalkiness. The excellent haplotypes of the *WCR1* [12], *OsAAP6*, [16] and *OsGluA2* [17] genes can be selected in salt-tolerant rice breeding.

## Data Availability

Data are contained within the article and Supplementary Materials.

## Supplementary Materials

The following supporting information can be downloaded at: https://www.mdpi.com/article/10.3390/ijms25074042/s1.
