# Supplementary material for: Moderate Salinity Stress Affects Rice Quality by Influencing Expression of Amylose- and Protein-Content-Associated Genes
Source: Int J Mol Sci. 2024 Apr 5;25(7):4042. doi: 10.3390/ijms25074042 (PMC11012469; doi:10.3390/ijms25074042)
Supplement: Supplementary file 1 [file ijms-25-04042-s001.zip › Table S1.pdf]

Table S 1. **Primers used for qRT-PCR of the genes used in this paper**

**Primer name**	**Sequence（5'-3'）**
eEF-1αF	TTTCACTCTTGGTGTGAAGCAGAT
eEF-1αR	GACTTCCTTCACGATTTCATCGTAA
qOsNF-YB1F	GAATATAGCGGCTCATCACC
qOsNF-YB1R	CACACACACATGCATCAAGTT
qWxF	ACCTGACACTGGAGTTGATTAC
qWxR	GTATGGGTTGTTGTTGAGGTTTAG
qChalk5F	TCCTTCACCCTCTTCGACTT
qChalk5R	GTGACGTAGCCTATCACCAATC
qOsAAP6	CAAGCGGAACTACACCTACAT
qOsAAP6	CCCAGAGAATCGCGTACTG
qOsGluA2F	TGCTTGTTCCTCTTGTGCGA
qOsGluA2R	TATGGCAACAACCGGCACTT
qWCR1F	CAGCCCATTCAGCCTCTTGCA
qWCR1R	CGGATTACCTCGACGAGCTC
